# A Qualitative Analysis of Clinical Year Veterinary Student Journal Entries for a Shelter Medicine Rotation

**DOI:** 10.3389/fvets.2022.858419

**Published:** 2022-04-06

**Authors:** Sohaila Jafarian, Eda Akpek, Chelsea L. Reinhard, Brittany Watson

**Affiliations:** ^1^VCA Veterinary Care Animal Hospital and Referral Center, Albuquerque, NM, United States; ^2^Mixed Methods Research Lab, University of Pennsylvania, Philadelphia, PA, United States; ^3^School of Veterinary Medicine, University of Pennsylvania, Philadelphia, PA, United States

**Keywords:** qualitative, shelter medicine, community-based research, veterinary student education, cultural humility, journaling, university, veterinary community medicine

## Abstract

Veterinary medical schools are tasked with not only providing experiences necessary to graduate veterinarians proficient in the skills and knowledge used most frequently in private practice, but also develop expertise in animal behavior, welfare, ethics, veterinary forensics, and cultural competency. Integrating all these knowledge areas into the curriculum can be challenging. Shelter medicine is increasingly identified by educators as an optimal learning environment to offer exposure to these topics. It can not only meet learning objectives in veterinary medical curriculum, but also provide students with authentic learning experiences to engage in and gain a deeper understanding of cultural humility, implicit bias, diversity, and inclusion. This includes documentation of international learning outcomes for shelter medicine in veterinary medical curriculum. There have been no studies evaluating authentic learning experiences specific to shelter medicine programs and their impacts on students. The aim of this study was to determine the individual activities and thematic categories on which veterinary students chose to reflect on and their potential impacts during a clinical rotation in shelter medicine at Penn Vet through qualitative content analysis of their journal entries. In our study, students found experiences on the shelter medicine rotation to be beneficial to their growth as a future veterinarian, provided them with takeaways that they found applicable and practical, helped them self-identify knowledge gaps, and changed their perspectives on several important topics related to collective human and animal welfare. These results speak to the value perceived by students of the rotation and suggest an authentic learning experience through a shelter medicine program can help cultivate more practice-ready and culturally competent veterinary graduates.

## Introduction

Veterinary medical schools are tasked with providing experiences necessary to graduate veterinarians who are proficient in the procedures, skills, and areas of knowledge used most frequently in private practice (1). Furthermore, it is evident that there is a need to provide graduates experiences in animal behavior, welfare, ethics, veterinary forensics, and cultural competency (2–16). Although gaps in veterinary education in these topics have been identified (2, 9, 16–20), veterinary medical schools continue to find it challenging to identify authentic experiences to integrate this curriculum.

Shelter medicine involves a breadth of topics critical to veterinary medical education that might be more challenging to incorporate into traditional training programs within small animal tertiary care facilities. Shelter medicine could be an optimal avenue to cover the care of the individual animal in the context of population management, herd health, public health, biosecurity, forensics, and high-quality high-volume spay neuter (HQHVSN). Additionally, shelter medicine provides the opportunity for students to engage with community outreach programs, learn about the dynamics of the inter-relationships of animal agencies within a community, how to serve as a resource between the veterinary community, the humane community, and the public, and to learn about access to care models (21–24). Moreover, shelter medicine programs can incorporate educational opportunities in animal behavior, welfare, ethics, community-based veterinary care, social justice, social work, animal abuse and the link with human abuse, and other topics relevant to collective human and animal health and welfare.

Due to its unique interdisciplinary public health content, shelter medicine has been utilized by educators as a critical tool in veterinary student education to provide experiences to fulfill accreditation standards established by the American Veterinary Medical Association Council on Education (AVMA COE). Additionally, in 2016, the AVMA COE expanded curriculum standards to ensure opportunities for students to “…gain and integrate an understanding of the important influences of diversity and inclusion in veterinary medicine, including the impact of implicit bias…” as well as the skills necessary for caring for the well-being of animals in the “context of ever-changing societal expectations” (11). Shelter medicine training can provide an authentic clinical experience that not only fulfills gaps in veterinary curriculum (25–32) but also provides service-learning opportunities (33–35) which creates an environment for students to engage in and gain a deeper understanding of cultural humility, implicit bias, diversity, and inclusion (36, 37).

Internationally, there are considerable differences between shelter medicine curriculums. Shelter medicine education abroad has traditionally been utilized to teach primary care skills, but is becoming more recognized as a subject worth teaching in it is own right (22). This recognition is apparent by the development of international learning outcomes for shelter medicine in veterinary medical curriculums which include the shelter medicine topics listed above (22).

The body of evidence available assessing the impact of shelter specific and service-learning experiences on students is growing, further supporting its value in filling the gaps present in veterinary curriculums. Research has shown that experiences in shelter medicine can increase perceived self-confidence and preparedness for real-world challenges in the field post-graduation (31) and increase self-confidence in various shelter medicine themes (29). These experiences have also been shown to increase student rating of their ability to perform six shelter medicine tasks as well as ovariohysterectomy/ovariectomy (OVH/OE) and castration surgeries (31), and decreased surgery time (28, 38) without a significant increase in complication rates (39). This finding of decreased surgery time without a significant increase in complication rates is further corroborated by studies in human medicine that have shown no difference between trainees and more-experienced surgeons in postoperative complication rates when careful patient selection and close supervision of trainees is applied (40–42).

Service-learning was a term first utilized in 1967, in reference to an internship program sponsored by the Southern Regional Education Board in which college students gained academic credit and/or federally funded financial remuneration for working on community projects (43). It was then further defined by three principles by Robert Sigmon in 1979 as (1) those being served control the service(s) provided (2) those being served become better able to serve and be served by their own actions (3) those who serve also are learners and have significant control over what is expected to be learned (43). The pedagogical practice of service-learning continued to grow and by the late 1980's it was clearly distinguished from community service by its attention to the integration of service with academic study (44). From the 1990s until today, colleges and universities have viewed service-learning as a central vehicle for fulfilling the commitment to civic responsibility (45). However, in more recent years, concerns about service-learning have surfaced from at least three separate large scale research projects (46–48). These concerns are rooted in that the dominant model of service-learning in the United States has historically focused on the education of students, rather than the achievement of community goals (49). A model for managing this shortcoming is community-based research (CBR). CBR emphasizes a research methodology where a collaborative community process determines the question, the research methods are designed to respect community ways of understanding, and the research results are connected to actual interventions (49). Other terminology and framework that might capture student learning based on real-life problems is an authentic learning structure. Authentic learning is a specific education term in literature that refers to multi-disciplinary, skills-based learning in a real-life context, demonstrating to students that their learning is connected, relevant, and can have an impact upon the world around them, as well as their future selves (50–52). Authentic learning is designed to connect what students are taught in school to real-world issues, problems, and applications. It is designed to help provide context and value to student learning, designed to mirror the complexities and ambiguities of real life (51). Authentic learning itself is inherently multidisciplinary, intentionally bringing into play multiple disciplines, multiple perspectives, ways of working, habits of mind, and community (52). It is also primed to provide students with the “portable skills” such as the flexibility to work across disciplinary and cultural boundaries to generate innovative solutions (53). This might be a more accurate description of the curricular approach in the shelter medicine field.

While there is a lack of research in veterinary medicine of the impact of CBR and service-learning projects, research documenting the impacts in human medical training is substantial. Studies in human medical fields have found that CBR projects and service-learning may impact professional and personal growth (54), increase confidence in one's ability to communicate effectively with their patients (55, 56), improve overall communication and interviewing skills (57), improve self-confidence and self efficacy (58), and enhance students' perceptions of their clinical reasoning skills (59–64). Additionally, facilitated discussion and reflective journaling are identified in literature as key to improving cultural competence in medical, pharmacy, public health, dental, and veterinary students (37, 65–70). There is also evidence that reflective skills and reflective practice seem to be essential for continuing personal and professional development in young veterinarians (71).

Despite the lack of research in the veterinary field, there appears to be growing interest among veterinary students and more recent graduates for working in the community (17). Moreover, there is a documented need for bringing more veterinarians into underserved communities and re-building trust between these communities and the veterinary field (18). A 2017 systematic review of the literature addressing veterinary care for underserved communities found that the most common barriers to veterinary care for vulnerable communities are cost, accessibility, veterinarian–client communication/relationships, cultural/language barriers, and lack of client education (17). Furthermore, this review revealed that when the barrier of communication and relationships between veterinarians and their clients were discussed, it was in the context of a lack of trust regarding cost, ethics, and judgment of the ability to offer care (17). Finally, this review suggests there is a care gap in veterinary medicine and animal welfare because clients do not understand the importance of and need for routine pet care (18, 72). While there is literature in veterinary medicine assessing service-learning programs utilizing shelter medicine (33–35, 38), and literature assessing service-learning's impact on veterinary students not specific to shelter medicine (36, 37), to the author's knowledge there has not been a study evaluating authentic-learning experiences specific to a shelter medicine program and its potential impacts on students.

Reflective journaling has been utilized as an educational tool for students in various human medical fields as well as a way to evaluate a program's efficacy through qualitative analysis (37, 65–70, 73–75). While most qualitative approaches seek to arrive at an understanding of a particular phenomenon from the perspective of those experiencing it, qualitative content analysis is appropriate when existing theory or research literature on a phenomenon is limited (76). Qualitative content analysis is defined as a research method for the subjective interpretation of the content of text data through the systematic classification process of coding and identifying themes or patterns (76). It is commonly used in healthcare research due to its structured and systematic process of analysis and can help identify the “lived experience” of groups (77). Lived experience, as it is utilized in qualitative research, is a representation and understanding of a research subject's human experiences, choices, and options and how those factors influence one's perception of knowledge (78). This approach also has the capability to understand phenomenon and gaps in research that quantitative research is inherently unable to appropriately investigate. This has been demonstrated in qualitative studies on medical students, revealing, for example, that medical students feared negative repercussions from disclosing mental illness and felt that they should be invulnerable to illness (79–81). Since this is the first-time clinical student journal entries have been analyzed for Penn Vet's shelter medicine rotation, and the desire to investigate the student perspectives, qualitative content analysis was utilized in this study (76). This process involves complete immersion into the data with no preconceived ideas or hypothesis from the researchers, allowing the meaningful themes to develop naturally into codes. A code in qualitative research is a word or phrase that summarizes or captures the essence of a portion of data (82). When utilizing qualitative data coding and analysis software, coding refers to gathering related material into a node, so when a node is opened one can see all the references in the text coded to that node (82). These nodes can then be organized into hierarchies moving from general topics at the top, referred to as parent nodes, to more specific topics referred to as child nodes organized under their respective parent nodes (83). To ensure systemic and subjective coding, the codes are then organized into a codebook to be referenced when coding the data. The codebook is substantiated through team-based qualitative analysis as described by MacQueen et al. (84) to ensure the researchers can assess the reliability and validity of the coded data through inter-coder reliability (85). Once the codebook is developed and established through inter-coder reliability, the data is coded, the nodes are quantified, and the data is analyzed.

## Program Description

The University of Pennsylvania's School of Veterinary Medicine (Penn Vet) is a 4-year professional program with the core portion of the curriculum extending over the first two and a half years and clinical rotations playing a major role in the remaining one and a half years. Students can participate in core and elective clinical rotations during the spring of third year and fourth year (86). Elective courses and a clinical rotation are offered by the Penn Vet Shelter Medicine Program. Since a strategic planning process was completed in 2015, the Penn Vet Shelter Medicine Program goals not only include traditional medicine and surgery, but emphasize wider sociological, economic, and cultural perspective with emphasis on representation of human-animal interactions and One Health concepts. This interdisciplinary approach utilizes authentic learning within the framework of CBR and service-learning models. The authentic learning framework was chosen for this rotation as it can provide learners the confidence that comes with being recognized as “legitimate peripheral participants” in a community (52). It can also help develop expert thinking which involves the ability to identify and solve problems for which there is no routine solution, and help develop complex communication skills such as gaining trust and building understanding (52). Moreover, there has been momentum within veterinary medical education to develop curricula that allow for authentic learning experiences (87).

The Shelter Medicine Program opportunities are provided in a service-learning model where students and faculty interact with the community. The program partners with nine different organizations, offering students a wide array of experiences within the field. These include open and limited admission shelters with varying intake numbers, as well as organizations that provide low-cost basic veterinary care and HQHVSN, participate in animal transport, promote community outreach, conduct forensic investigation and humane law enforcement, and/or host behavior programs. Other collaboration includes humane education opportunities with local middle and high schools. Although the clinical rotation focuses on the shelter animal medicine field, students also gain skills and experiences that are valuable in various types of practice including small animal clinical medicine, public health, behavior, community-based veterinary care, and forensics (88).

The activities students participate in during the clinical rotation include surgery, physical exams, home visits, tours of shelter partner facilities, student presentations, and targeted consultations. In addition to these activities, rounds topics include biosecurity, behavior, risk analysis, exotic animal care in the shelter environment, animal welfare and ethics, humane law enforcement, and “real life” rounds referring to contract negotiation, work life balance, and direct student feedback (Table 1). Finally, there are two assignments required to be completed by students during the rotation: an example social media post and journaling. The written social media post is utilized to highlight the importance of social media in promoting animals for adoption by animal shelter organizations. For the journaling assignment, students are required to submit two journal entries that ask the student to reflect on their experiences during the rotation. They are verbally notified on the first day of the rotation by service faculty that the faculty will read the entries, which are submitted non-anonymously and that excerpts from the journal entries may be shared but any shared portions remain anonymous. Only the submission of the two required journal entries is required for the rotation; the content of the entries has no bearing on the student's grade for the clinical rotation. The writing prompt for the journal entries is posted online as follows, “Please create two journal entries based on your experiences during the rotations. You can focus on something that surprised you, a case that inspired you, someone you met that changed your perspective on things, an experience you particularly liked or didn't like, an ethical issue you want to expand on, etc. We want you to reflect and for us to understand what is meaningful to you during the rotation experience. These entries should be a minimum of 500 words and submitted as two separate documents.”

**Table 1:** Details of each activity the students participate in and additional detail on topics for rounds held on rotation.

Activities and rounds topics^*^on rotation	
	**Description**
**Activity**	
Surgery at high quality high volume spay/neuter clinic	The purpose is to expose students to a fully staffed HQHV spay-neuter clinic and to gain spay-neuter surgical experience
Surgery at large open admission municipal shelter	Students will perform spay-neuter surgery in the shelter setting. Additional surgeries or other opportunities such as necropsies are possible as they come into the facility
Physical exams	Students perform hands on veterinary walk-throughs and exams on patients. Students will apply risk analysis techniques, herd health, and individual animal welfare/health at various shelter partner facilities. Creating treatment plans in the shelter setting is discussed
Home visits	Home visits are done through the shelter program's community outreach partner. The community outreach program is based on preventing animal surrender at the most at-risk areas by surgical, medical, and educational interventions. Students will provide some basic medical care but focus on creating trust and relationships in at risk animal populations in door-to-door outreach. Hands-on and communication skills are emphasized
Facility tours	Students tour all shelter partner facilities
Staff rounds	Students participate in topic rounds, receive mentorship, and debrief on experiences
Student presentations	Students will present humane education information to an area middle or high school. They will think about teaching to an audience. When available they will help design a lesson plan and outreach for students in the area. Students will give CE presentations to shelter medicine staff. This demonstrates the skills and knowledge they have learned about shelter medicine, education, and communication. It also shows students' ability to gather topic specific information independently. Debrief on the presentation will occur immediately following
Targeted consults	Not routinely held, only when a partner facility needs a targeted consultation. When a targeted consult is needed, students create a product that usually involves giving advice or recommendations to the shelter
Rounds topics^*^	
Biosecurity rounds	Students review concepts of biosecurity in the shelter setting and perform hands on veterinary walk-throughs and exams on patients. Students apply risk analysis techniques, herd health, and individual animal welfare/health at the largest open admission shelter in Philadelphia. Creating treatment plans in the shelter setting is discussed
Behavior rounds with Penn faculty	Students cover body language, surgical pain scoring, and behavioral assessment in the shelter setting
Behavior rounds with shelter partner	Students use the knowledge of training, body language, socialization, and behavioral evaluation on-site with a certified behaviorist
Risk analysis rounds	Students complete a risk analysis chart during exotics rounds
Exotic animal rounds	Students evaluate shelter exotic animal housing and complete a risk analysis chart. Discussion on ethics will be emphasized
Animal welfare and ethics rounds	Students discuss the complex issue of feral cat management and the ethical implications. This is also an opportunity to discuss any other animal-related ethical issues
Humane law enforcement with shelter partner with forensic programs	The shelter partner focuses on their law enforcement mission including possible time with veterinarian, lawyer, and officer whose primary purpose is in this field
Real life rounds	Real-Life rounds discuss important lifestyle issues including compassion fatigue, burnout, managing stress, contract negotiation, and the similarities between private practice and shelter medicine stressors

* *This is meant to be representative of rounds typically held during the rotation*.

This is a foundational qualitative investigation to assess how authentic learning experiences on a shelter medicine rotation impacts students, identify potential research gaps in shelter medicine education, and to help guide further research. The rationale for this study was to determine the individual activities and thematic categories on which veterinary students choose to reflect on during and their potential impacts during a clinical rotation in shelter medicine through qualitative content analysis of their reflective journal entries.

## Methods

This retrospective study examined completed journal entries from third and fourth year veterinary students participating in the shelter medicine clinical rotation at University of Pennsylvania from January 2015 to July 2019. All entries accessible by the researcher in the fall of 2019 during this time period were included in the qualitative analysis. Journal entries excluded from the analysis were those entries submitted outside of these dates, journal entry files that were corrupt, or journal entries that had lapsed access permissions (total excluded entries *n* = 42). In total, there were 200 total journal entries analyzed, representing 106 students. The discrepancy in the ratio of journal entries to students is due to factors such as students submitting both journal entries under one file with each file counted as one entry, students taking the elective more than once, and students only having only one accessible journal entry. The journal entries were analyzed retrospectively *via* qualitative content analysis (76). This process involves developing a theory based on the data rather than data collection being a process of testing a pre-existing theory (89). This process of data analysis involves a series of systemic cycles of inductive elaboration, deduction, and verification (90).

Data analysis began with the one researcher (SJ) reading all the journal entries to achieve immersion and obtain a sense of the whole (85). The researcher (SJ) made notes of their first impressions, thoughts, and initial analysis and during this process initial codes emerged. These codes were then sorted into different categories based on the structure of the shelter medicine rotation and previous knowledge of shelter medicine using NVivo 12, a software program for qualitative data coding and analysis (91). Once this initial coding process and saturation of themes was accomplished, the secondary researcher (EA) independently coded 20% of randomly selected journal entries and compared for inter-coder reliability. Inter-coder reliability was calculated with NVivo 12 by using Cohen's kappa (k) coefficient (92). Complete agreement in coding correlates with a mean k of 1; near perfect agreement, a mean k of 0.81–0.99; substantial agreement, a mean k of 0.61–0.80; and moderate agreement, a mean k of 0.41–0.60. Discrepancies in coding were resolved with reviewing and consensus on adjustments to the codebook. During this review process, it was decided that to address our research questions and maintain clarity through the coding process the parent nodes would be split into activities, location, shelter medicine, student perspectives, and syllabus. The parent nodes of activities and location were meant primarily to answer the first research question of which activities students reflected on and in what frequency. The parent nodes of syllabus and shelter medicine were designed to capture larger concepts across the rotation that students chose to reflect on and utilized to gain a deeper understanding of the student perspective themes. Finally, the parent node of student perspectives arose solely from thematic content analysis of students' reflections on their experiences during the rotations. Once the review process was completed, the secondary researcher (EA) independently coded 20% of randomly selected journal entries once again and compared for inter-coder reliability. A mean weighted Cohen's kappa of 0.80 was achieved, the codebook was finalized, and the remainder of the journal entries coded by the primary researcher (SJ). The full codebook with descriptions of each parent node and child node is provided in Appendix 1.

This study was determined to be exempt from the University of Pennsylvania's Institutional Review Board (IRB #4) review after completing the Human Subjects Research Determination Form (#843548).

## Results

A total of 200 journal entries were evaluated in this study from 106 clinical students. The coding was organized into the five broad parent node topics: (1) activities, (2) location, (3) shelter medicine, (4) student perspectives and (5) syllabus. The codes that were assigned to these topics represented: (1) activities that the students participated in as part of their learning, (2) locations that the students visited, (3) any themes identified relating to the realities or core concepts of shelter medicine (not including those that fell under Syllabus), (4) student perspectives in which included themes derived directly from content analysis of the journals, and (5) core topics or lessons from the course syllabus (specifically the “learning opportunities” descriptions) students discussed in their entries. The quantified results of mentions of each node, organized by parent node, are in Table 2.

**Table 2:** Quantification of the total journal entry mentions for each node, the percentage of journal entry mentions for each node as a percentage of the total journal entries, and an approximation of hours spent on each activity throughout the rotation.

	**Total entry mentions**	**Total percentage of entry mentions %**	Hours on activities^*^
**Activities**			
Physical exams	86	43	12–14 h^**^
Home visits	50	25	6–8 h
Facility tours	46	23	6 h
Surgery	46	23	22–32 h^***^
Staff lectures	38	19	6–9 h
Student presentations	22	11	2–4 h
Targeted consults	4	2	Varies by rotation
**Location**			
Large open admission municipal shelter	94	47	
Community outreach organization	59	29.5	
Shelter that conducts forensic investigation and humane law enforcement	51	25.5	
Small open admission shelter	23	11.5	
Shelter with behavior program	19	9.5	
Middle/high school (location varies)	16	8	
Shelter with low-cost basic veterinary care and HQHVS	14	7	
Shelter with HQHVS	12	6	
Philadelphia's largest rescue partner, also provides low-cost basic veterinary care	11	5.5	
**Shelter medicine**			
Limitations	100	50	
Euthanasia	54	27	
**Student perspectives**			
Beneficial experience	133	66.5	
Changed perceptions	119	59.5	
Takeaways from activities	108	54	
Human animal bond	47	23.5	
Judgement	34	17	
**Syllabus**			
Community outreach	74	37	
Shelter operations	72	36	
Behavior	70	35	
Spay and neuter	70	35	
Animal welfare—ethics	66	33	
Real life	49	24.5	
Law enforcement	43	21.5	
Biosecurity	36	18	
Feral cat management	20	10	
Exotic animal	14	7	
Dental	3	1.5	
Pain scoring	0	0	
Public health	0	0	
Risk analysis	0	0	

* *Hours spent on an activity vary occasionally due to factors such as availability of surgical candidates, holidays, facility matters, etc. The hours listed here are meant to provide an idea of a typical rotation schedule*.

** *Physical exams were not divided into which location the activity took place as there were numerous students reflecting on the activity and not the location. Majority of physical exams were performed at the large open admission shelter, however opportunities do arise for physical exams at other shelter facilities during the rotation. The exception is home visit physical exams which were their own node*.

*** *Surgery mentions were not divided into which location the activity took place as there were numerous students reflecting on the activity and not the location*.

As discussed in the methods section, the parent node of student perspectives was derived solely from content analysis of the journals. The most common themes manifested were: (a) beneficial experiences, (b) takeaways from activities, (c) changed perspective, (d) human-animal bond, and (e) judgement. All five of the student perspective themes were the most frequently mentioned and will thus be the focus of the results, in the context of which activities were mentioned in correlation with each student derived theme.

### Beneficial Experience

Beneficial experience was the most common student theme, with 133 out of 200 journal entries (66.5%) describing one or multiple experiences on the rotation to be beneficial. This theme was identified by student journal entries describing an experience during the rotation was beneficial to their growth as a veterinarian, helped prepare them for clinical practice, added to their skillset, helped them gain confidence, or aided in overcoming an obstacle (such as previously held fears or anxieties). Students reported that their experiences during the shelter medicine rotation that are not offered through the core curriculum or other clinical rotations were beneficial.

The activity most mentioned with beneficial experience was physical examinations, with 53 journal entries (43%) describing physical examinations to have been beneficial. Surgical experience was also regarded as beneficial, but less frequently than the physical exams with 31 journal entries (23%). Students discussed multifocal benefits of shelter facility walk-throughs, physical exams, and developing diagnostic and treatment plans. They related these experiences to more “real world” veterinary medicine, managing conditions they would expect to see once in practice. They reflected on experiencing more routine cases and navigating a diagnosis without the advantage of unlimited diagnostics. Learning opportunities discussed by the students included critically evaluating cases, conducting thorough physical exams, discussing differential diagnoses and rule outs, available diagnostics and the value of each, treatment options, patient monitoring in treatment plans, and expected outcomes. Students expressed the benefit of learning a spectrum of care and found it to be practical to prepare for practice.

“While not every diagnostic test was available, I learned the value of a truly thorough physical exam. For instance, when examining a case of diffuse hair loss in a canine, [the faculty instructor] used [their] keen observational skills to note that there was crusting and abrasions cranial to the tail head. This allowed for the prioritization of the differential diagnoses list, potential diagnostics and treatment options. Also, with limited resources, [they were] able to rank order potential treatment options. There was a stronger emphasis on the future monitoring protocol to allow tailoring of the treatment plan as needed over time. This is crucial when considering a conservative line of treatment … It was also during this day that I realized that while there may be a documented ‘gold standard' of treatment, it feels more apt to characterize veterinary medicine as a ‘spectrum of care' that all fit within the accepted standards of care…I hope to be able to provide a spectrum of care for my patients and learn to prioritize potential diagnostic/treatment options.”

Students reflected on the importance of stepping outside of the academic environment for more real-world experience. As one student wrote,

“The shelter medicine rotation was one of the most valuable experiences I had at Penn. It afforded me the privilege of experiencing some incredibly relevant, real-life veterinary medicine which is so rare in the rest of our curriculum. At a University that is so academically focused, the opportunity to step outside and interact with the non-academic people at the shelter partner locations is vitally important to a complete education.”

Students also reflected on the immense benefit of mentorship throughout the rotation, including physical exams, surgery, and other hands-on experiences, along with learning the destructive nature of perfectionism that can be prevalent in the field of medicine. They found that the rotation provided a kind of mentorship they had not been exposed to previously, an open and constructive learning environment where students could gain confidence. Students found that medicine requires lifelong learning, and this perspective is one they found encouraging.

“I have observed a remarkable transformation in myself in just the four surgery days on the Shelter Medicine rotation. The three surgical days that followed the first challenging day were key in the development of my surgical skills and growth in confidence. Not only have I become tremendously more relaxed about surgery, but I have also realized that I can think around small challenges that arise during the procedure… The mentorship, encouragement, and constructive feedback you provided while we spayed and neutered was essential in how much I grew in just 4 days (And for that I thank you!). The surgery experience was also a lesson for me in patience. On the coattails of a difficult previous rotation when I deeply questioned my competence, I was quick to criticize my skillset. But after the first surgery day on Shelter Medicine, I continued to spay and neuter, and with a new goal: to be patient with myself and my learning. The process of giving myself space to learn, along with the right teaching, was ultimately what resulted in a much more confident and patient student of surgery.”

### Takeaways

The student theme of takeaways was the third most common theme, with 108 out of 200 journal entries (54%) expressing this theme. This theme captured students reflecting on how they will take what was learned on the rotation and utilize it in their career. There was significant overlap between the themes of beneficial experiences and takeaways. However, when exploring the takeaways theme, it was evident that students felt empowered by their experiences during the rotation. They were exposed to tools such as humane education and behavior training that they found applicable and felt they could realistically incorporate into their practice. Students also reflected on more abstract tools such as being provided hope for situations they previously considered hopeless. Students expressed gratitude for being provided these tools prior to graduation and to have the opportunity to re-evaluate their role in their community.

“I appreciated having the opportunity to not practice “gold standard medicine.” It was nice to step out of the ivory tower… and practice formulating plans and diagnostics that do not require a lot of resources and money. I think we forget how much can be done without an ultrasound for everything! I'm often worried about making the transition from [academia] to general practice, and this rotation really helped me practice my “real world” approach and sharpen my tool kit.”

Additionally, many students reflected on situations that they had previously thought were “hopeless.” Not only did these students see potential for positive change after their rotation, but also saw how they could be a vehicle for that change. Students found that they were an integral part of their community, and upon discovery of this fact felt deeply empowered.

“I feel so passionate about animals but time and time again I feel a bit hopeless. I want to make a difference in animal welfare but its always hard to know where to start. This rotation has definitely allowed me to appreciate that it is possible. People work at it everyday and even small steps make a huge difference to these animals, which only serves to motivate me.”

“It is nice to know that we helped someone simply out of kindness, but it is also a great example of how veterinarians can utilize more than just the practice of medicine to improve animal welfare…It is a good feeling to know that our knowledge and abilities as veterinarians can not only help animals, but also earn the trust and strengthen the connection with a part of our community that is sometimes forgotten.”

Students also reflected on the power of humane education, and how to present information and build trust and connection with people of varying backgrounds, cultures, beliefs, and education levels. This result was especially impactful considering students only spend roughly 4–6 h on the rotation presenting humane education information in the form of continuing education presentations or during home visits. They reflected on this being an avenue for positive change they did not realize existed and one that made them feel like a more important figure in their community.

“As we drove away, we discussed with [faculty instructor] how such a simple in home appointment and basic education on pet health care prevented a dog from ending up in the shelter and an otherwise caring pet owner from experiencing the pain and guilt of surrendering [their] beloved animal. We talked about how this type of outreach to underserved communities is integral to increasing owner retention, which in turn, decreases the number of animals ending up in the shelters. This was a role of a shelter veterinarian I had not fully understood until now.”

“… I love the ability to discuss veterinary medicine with people. I really enjoyed the outreach at the high school and the opportunity to talk about one of my favorite subjects. … I think it is important for students to learn how to speak to people with different backgrounds since this is a more accurate representation of who they might work with in private practice. I love the idea of providing continuing education talks at shelters, as suggested by [faculty instructor]. It provides me the opportunity to share my interests of exotics and behavior with others. Overall I enjoyed this rotation very much and have learned new ways to work with shelters after graduation.”

Of the overarching concepts that students discussed as providing them takeaways, the most common was behavior, with 36 journal entries (18%) mentioning both takeaways and behavior. Students reflected on the behavior activities and how hands-on training in behavioral evaluation, interventions, and socialization provided students tools they could take with them to practice. Students found this experience to be novel and discovered many new skills that they found useful for practice.

“While the behavior rounds were helpful in reviewing content we've covered in previous classes, the behavior session at [partner location] was some of the most helpful behavior material we've received so far in the curriculum, in my opinion. We've had multiple lectures covering body language, the signs of stress, specific displacement behaviors, and the quadrants of conditioning, but after a certain point it seemed like each one mainly covered warning signs to look out for in dogs and not as much about applying it. Being able to apply that background knowledge in different training sessions was immensely useful so that we could get some practice with the process of training, learn potential pitfalls, and get some tips on how to adjust”

“I learned a lot during this rotation, but I enjoyed ending this rotation with behavior evaluations. I felt that this was particularly applicable to my future career, as well as to animals in shelters. Behavior and training are very important parts of pet ownership and it was great to have some hands-on exposure to behavior examination and evaluation during this rotation.”

### Changed Perceptions

The student theme of changed perceptions included any mention by a student regarding a perception they had on any topic, including but not exclusive to shelter medicine, that was changed by their experiences during the rotation. The most common experiences leading to these changed perceptions were grouped into: (1) animal welfare/ethics/law, (2) shelter operations and resource limitations, and (3) their experience with the community outreach organization. Within these three broad topics students also self-identified significant knowledge gaps that they were previously unaware of.

There was a total of 74 journal entries (37%) that mentioned changed perceptions, animal welfare/ethics, and humane law enforcement. Students reflected on the lack of defined laws in regards to animal abuse, the difficulty of prosecution in suspected animal abuse cases, the long legal hold times involved in cruelty cases, the extent and prevalence of animal abuse, appropriate management of a suspected case of abuse, and that some cruelty cases are not clear cut intentional cruelty but rather due to circumstances such as lack of education. While many students grappled with these realities, a majority felt inspired and empowered by the time spent with forensic shelter staff and the skills learned. Many wrote that they wished it were incorporated into the core curriculum and were grateful to have been exposed to these concepts prior to graduation.

“There were many aspects of [partner veterinarian's] work which had never crossed my mind like how to properly document abuse and prove incidences o[f] cruelty. It was difficult to hear about the cases [they] saw because I don't want to believe that people do such horrific things to animals. I was also not aware about how important cruelty cases can be from a justice point of view … Since there is a link between animal abuse with child abuse and domestic violence I can see how cruelty cases are of extreme importance both for the sake of the animals but also for other potential human victims involved.”

“…Ultimately, I enjoyed our visits to [shelter partner]. Though I was upset by many things I learned, it was all informative and made me think critically about laws surrounding animals, this shelter's individual practices, and animal ethics and welfare in general.”

There was a total of 91 journal entries (45.5%) that discussed changed perceptions, shelter operations, and limitations (referring to resource limitations encountered in the shelter setting). Some students were unaware of how many different kinds of shelters there are, how they work together, how shelters are an integral part of their community, how much shelters can do, and the realities behind many highly stigmatized topics such as “kill vs. no-kill” status, trap-neuter-return programs, and intake policies. Once again, although students grappled with the challenges faced by the shelter communities, they were also empowered by the knowledge, discussions, and shelter staff.

“I was grateful that our time at a variety of Philadelphia shelters was extensive enough to appreciate some of these differences between shelters. We regularly debriefed or rounded as a group at the shelters, discussing unique shelter challenges and veterinar[y approaches] within the shelter medicine field. Our rich discussions and shelter tours have exposed me to a side of veterinary medicine I had not previously considered. I am thankful that I am more informed about the field of shelter medicine, as it will enable me to better serve clients and work cooperatively with companion and shelter animal veterinarians.”

Finally, students reflected on the way community outreach impacted their views, which also encompassed student themes of human-animal bond and judgement. In total, there were 43 journal entries (21.5%) with the themes of changed perceptions in the context of community outreach, with 33 of those entries (76.7%) being specific to their experience with the community outreach program. A majority of student journals reflecting on the two student themes of human-animal bond and judgement were in relation to their experience in the community outreach program, with 38 and 25 journal entries (61 and 54%), respectively. Despite only spending ~6 h of the rotation with this community outreach program, this experience changed students' perspectives on this community of pet owners, helped them gain a deeper understanding and appreciation of the human-animal bond, helped them gain insight on the realities of veterinary deserts, helped them gain understanding on the detrimental impact of judgement and implicit bias, and helped them see their own previously unrecognized implicit bias. Students found this experience deeply impactful and reflected on how important an experience like this is for a field that is severely lacking in diversity.

“Veterinary medicine is a great field—but the lack of diversity within the profession is staggering. There is a palpable discomfort that most students have simply talking about… neighborhoods that live below the poverty level in Philadelphia—let alone engaging in positive interactions with people whose initial reaction to topics like spay and neuter is vastly different from a typical Penn Vet student. For that reason, [community outreach organization] doesn't just serve as a resource for people and their animals in these veterinary care deserts—it also serves as an eye awakening experience for students into where the profession often fails.”

### Human Animal Bond

As mentioned, students' reflection on the human-animal bond was overwhelmingly in relation to their experience with the community outreach program, with 38 out of 62 of journal entries (61%) discussing this theme in relation to their experience with the program. Students wrote about the impact it had on them to see just how deeply the people of these communities love their pets, many seeing their own love of animals in the interactions on these home visits. Students realized how well-intentioned owners are and that many have spent a lot of money on their pets, but that money was spent on treatments that may not have been appropriate for the issue their pet was suffering from. Students were unaware that these treatments were often due to misinformation and in some cases the misinformation came from other veterinarians, further eroding the trust between veterinarians and this community. Students found this community of pet owners had a fierce sense of commitment to their pets, had high levels of owner compliance, and looked to the students for affirmation that they are “doing a good job” as pet owners. These interactions led many students to reflect on their own “right” to own an animal, leading many to come to the conclusion that the emotional, mental, and health benefits of the human-animal bond should not be limited to owners with unlimited resources.

“The people served by [community outreach program] LOVE their pets. They would often times do anything for them and simply due to limitations such as transportation and finances, the care provided is not as good as they would [like]… But as someone who has pets and knows firsthand the incredible benefits of having pets in [their] li[f]e, I think it is important to take into account how many emotional and mental benefits are brought to people's lives every day as a result of owning a pet.”

“Another interesting aspect of riding around with [community outreach program] was how clear it was that the owners cared for their animals, yet if these animals walked in through the doors at [tertiary care hospital] there may have been some judgment made about them. Some of the animals weren't in the best of condition…but it was very clear the owner truly cared for her dog and was doing the best that she could to make sure that her dog was getting the best care the[y] could considering her circumstances.”

### Judgement

Finally, students reflected on the concept of judgement within the field of veterinary medicine and overwhelmingly in the context of their experience with the community outreach program with 25 of 46 (54%) of journal entries discussing this theme in relation to their experience with the program. Students noticed not only a shift in their perspective, but when interacting with a new client to this community outreach program, they noticed the shift in the owner when the owner realized they were in a safe, non-judgmental environment. This led many students to realize that not only does this cohort of pet owners communicate their love for their pets, but that they may feel judged by the veterinary community when reaching out for help. This experience led students to recognize they had misplaced judgement and implicit bias against this community, with many stating, “Who am I to judge?” Students reflecting on judgement came to the realization that recognizing their own implicit bias was something they would have to continuously reassess within themselves, and that they would strive to be a resource and build trust in underserved communities.

“Several people we visited took in animals off the street that needed help or went to adoption centers to pick up pets that may not have been adopted otherwise. I'm starting to see that these animals have a better quality of life with these loving owners that have limitations on veterinary care than they would in the streets or waiting in numerous shelters. Just because people can't afford an emergency room visit doesn't mean they don't care. It could just mean they were being a good Samaritan [and] are looking for resources and care for animal they've rescued from off the street. It's important that I not judge certain people too quickly; they are animal lovers [just] like myself who are looking for a meaningful bond between themselves and an animal just like I am.”

“My experience with [community outreach program] really changed my perspective on pet ownership in under-served communities. Before encountering this program, I admittedly had a rather close minded view about people owning pets without the means to support them. I thought that if you are going to adopt an animal, you should only do so if you are financially able to be responsible for that animal. I was jaded by my experiences… However, my opinion completely changed after my experience with [community outreach program]. This program convinced me that people in the under-served communities can be great pet owners and that they deserve the opportunity to have their own pets.”

### Summary Figure

**Figure 1:**
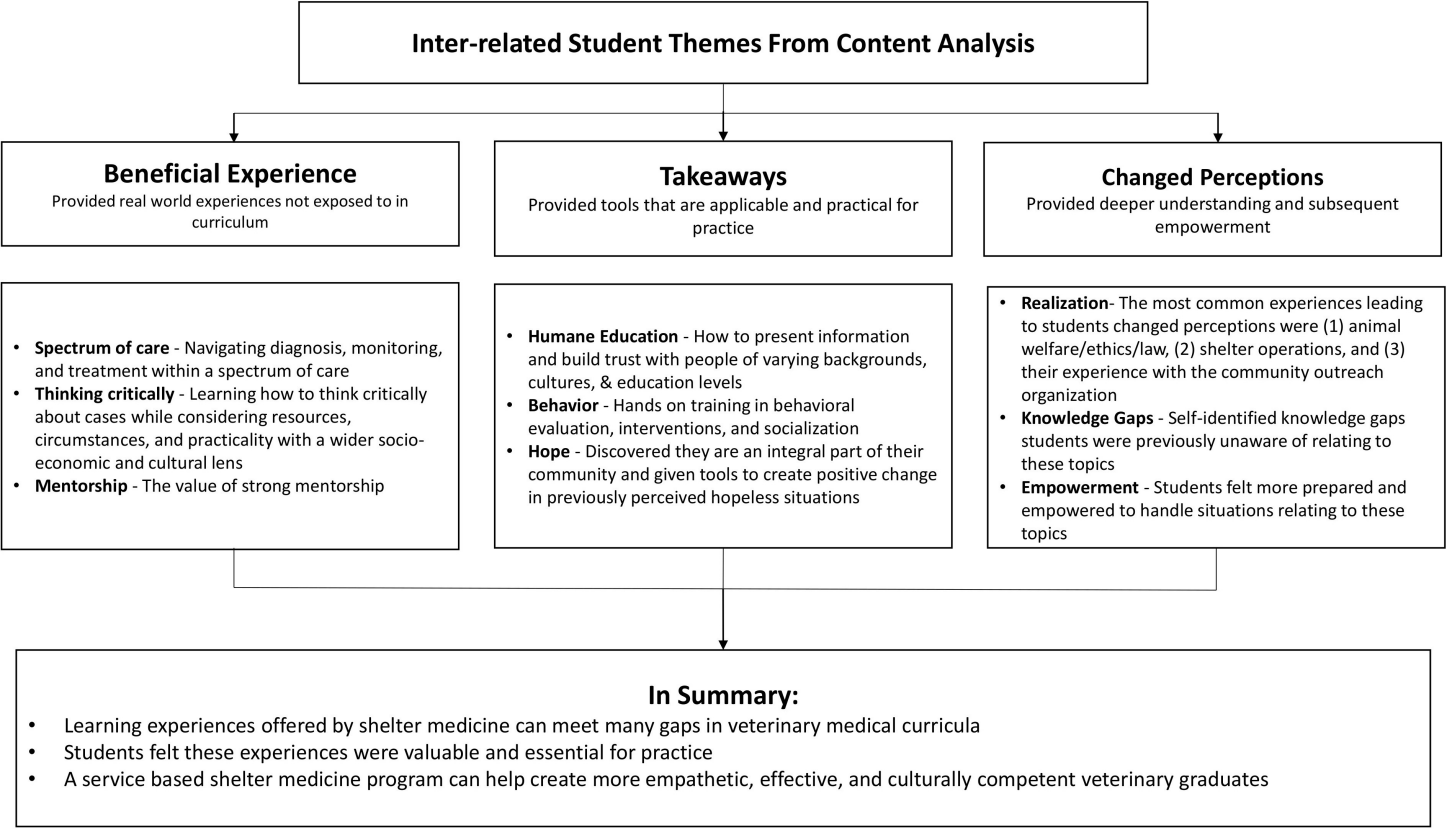
A summary of the three inter-related themes that emerged from the content analysis.

In summary, the inter-related themes that emerged from the content analysis were beneficial experience, takeaways, and changed perceptions (Figure 1).

## Discussion

The authentic learning experiences offered by shelter medicine can meet many gaps in veterinary medical curricula and is also a critical competency for veterinary graduates (22). To our knowledge, this is the first paper utilizing qualitative analysis to gain a veterinary student perspective on authentic learning experiences and their impacts during a shelter medicine rotation. This research is a foundational piece to assess how authentic learning experiences on a shelter medicine rotation impacts students, identify potential research gaps in shelter medicine education, and to help guide further research. Qualitative content analysis was chosen as the research methodology as it is the most appropriately designed to explore data in which research is limited as well as having the potential to identify gaps in research that have been previously unidentified. Gaps in research and understanding are inherently more difficult to identify with a traditional quantitative approach, which has been demonstrated in this study and in studies on lived experiences of medical students (79–81). Evaluating student learning experiences through this type of qualitative model needs to be considered to allow for a better understanding of the full context of student learning in academia. This is particularly important in complex clinical settings where interpretations of experiences and interactions can be critical for growth and learning of the student body.

Traditionally, universities have focused their shelter medicine educational research on surgical skills gained on rotation. While there is validated benefit in these surgical experiences (28, 31, 39), this study indicates that there are more experiences in shelter medicine education that students find deeply fruitful which warrant further investigation. This is especially evident considering students spend roughly 1/3 of their time on the rotation performing surgery, yet it was the 19th most common mention in our journal entries. This was an unexpected finding considering that anecdotally schools report value associated with surgical experience. The surgery mentions fell behind less traditionally notable topics such as physical exams, community outreach, shelter operations, behavior, and animal welfare/ethics/humane law. In our study, students found these experiences on the shelter medicine rotation that are not a part of the core curriculum to be beneficial to their growth as a veterinarian, provided them with takeaways that they found applicable and practical, helped them self-identify significant knowledge gaps, and changed their perspectives on several important topics. They valued learning how to think critically about cases while considering resources, circumstances, and practicality with a wider socio-economic and cultural lens. They found that the experiences made them feel more prepared for practice and provided them with practical and applicable tools which they felt was lacking in the core curriculum. These results speak to the gaps in the core curriculum, the need for more research into the traditionally less notable topics within shelter medicine, and the value of these topics perceived by students.

There were many circumstances related to animal welfare and humane law enforcement, resource limitations within the shelter setting, and vulnerable communities that students revealed they now felt more equipped to handle. They reflected that previous knowledge and experiences had led them to believe these situations were hopeless. During this rotation, they were introduced to the reality that veterinarians are an integral part of their community and that they could create positive change in animal and human health and welfare. This was a role that students did not expect to play in their career and were unaware of the change they could make in their communities. Students were surprised by the power of humane education and how much they could do outside of traditional clinical skills, which led them to realize the knowledge they had was a valuable tool in and of itself. They found utility in learning how to present information and build trust and connection with people of varying backgrounds, cultures, beliefs, and education levels. They found this to be an avenue for positive change they did not realize existed and some even wrote about continuing humane education outreach in their career. This study indicates that exposing and educating students on difficult topics within the authentic learning framework has the potential to change perspectives and empower students on these topics and warrants further investigation.

There is ample literature demonstrating that facilitated discussion and reflective journaling has been found to be key to improving cultural competence in medical, pharmacy, public health, dental, and veterinary students (37, 65–70). There is also evidence that reflective skills and reflective practice seem to be essential for continuing personal and professional development in young veterinarians (71). We found that these experiences, facilitated discussions, and reflective journaling not only impacted students' cultural competence, but also helped develop cultural humility and awareness of their own implicit bias. A 2017 systematic review of the literature addressing veterinary care for underserved communities found that the most common barriers to veterinary care for vulnerable communities are cost, accessibility, veterinarian–client communication/relationships, cultural/language barriers, and lack of client education (17). Furthermore, this review revealed that when the barrier of communication and relationships between veterinarians and their clients were discussed, it was in the context of a lack of trust regarding cost, ethics, and judgment of the ability to offer care (17). Finally, this review suggests there is a care gap in veterinary medicine and animal welfare because clients do not understand the importance of and need for routine pet care (18, 72). This study shows that an authentic learning shelter rotation could help produce veterinarians that are more equipped and empowered to help overcome these barriers for care and re-build trust of the veterinary community and validates further research.

The limitations of this study were due in large part for the desire to capture mentions of all activities, syllabus learning objectives, and locations. This led to an excessive number of nodes and thus leaving few nodes for student themes. While each student theme was explored, we were unable to fully explore these themes to the full extent within this paper. Additionally, the responses around the community outreach activities, humane education, and the community outreach program were so numerous and diverse that we were unable to fully capture within the confines of this paper. The community outreach experiences will be the focus of future evaluation of these journals, due to the impact demonstrated within the entries. Although this paper covers a foundational overview of the rotation, the journal coding can be used as a foundation for more in-depth exploration in future studies. Additionally, although many students appeared comfortable discussing difficult topics, students may have altered their responses knowing that their instructors would be reading their journal entries. There are also limitations within qualitative research, in that it is inherently more subjective that quantitative studies, which can lead to error and bias (77).

## Conclusion

In this retrospective qualitative content analysis research study, it was identified that the Penn Vet Shelter Medicine Rotation students chose to reflect on the value of the rotation experience surrounding themes of primary care experience, humane education, behavior, animal welfare/ethics/law, shelter operations, community outreach, human-animal bond, and judgement. This suggests that a shelter medicine rotation based in an authentic learning framework can contribute to the required veterinary school curriculum within the United States and internationally in a meaningful and impactful way for students. Providing authentic learning experiences with community outreach, collaborative partnerships, and service-learning in a variety of organizations within the surrounding community provided a deeper and more diverse experience of veterinary medicine than what is experienced in the core curriculum. This, along with the mentorship, facilitated discussions, and reflective journaling revealed that students felt empowered and changed their perspectives on vital topics. Considering these results in the context of the current political climate, an authentic learning shelter medicine program can help cultivate more empathetic, effective, and culturally competent veterinary graduates.

## Data Availability Statement

The raw data supporting the conclusions of this article will be made available by the authors, without undue reservation.

## Ethics Statement

The studies involving human participants were reviewed and approved by University of Pennsylvania Institutional Review Board. Written informed consent for participation was not required for this study in accordance with the national legislation and the institutional requirements.

## Author Contributions

SJ, EA, BW, and CR contributed to conception and design of the study. SJ read all journal entries and developed initial codes and achieved saturation of themes, coded all journals once codebook was finalized, and wrote the first draft of the manuscript. SJ and EA coded data independently, compared coded data to develop and refine the codebook, and finalized the codebook once appropriate kappa agreement was met. All authors contributed to manuscript revision, read, and approved the submitted version.

## Funding

Salary for SJ position and this research was supported by the Arnall Family Foundation. Salary for CR was supported by the Bernice Barbour Foundation.

## Conflict of Interest

CR and BW were course organizers for the shelter medicine rotation. The remaining authors declare that the research was conducted in the absence of any commercial or financial relationships that could be construed as a potential conflict of interest.

## Publisher's Note

All claims expressed in this article are solely those of the authors and do not necessarily represent those of their affiliated organizations, or those of the publisher, the editors and the reviewers. Any product that may be evaluated in this article, or claim that may be made by its manufacturer, is not guaranteed or endorsed by the publisher.
