# Supplementary material for: A Qualitative Analysis of Clinical Year Veterinary Student Journal Entries for a Shelter Medicine Rotation
Source: Front Vet Sci. 2022 Apr 6;9:858419. doi: 10.3389/fvets.2022.858419 (PMC9019659; doi:10.3389/fvets.2022.858419)
Supplement: Supplementary file 1 [file Data_Sheet_1.docx]

**Appendix 1**

The finalized codebook utilized in coding journal entries; organized by parent node (bolded and underlined) and node.

| Codebook | |
| --- | --- |
| **Name** | **Description** |
| **Activities** | Activities that students participated in as part of their learning |
| Staff lectures | Student discusses rounds, lectures, mentorship, direct or group feedback, or any other comment by “staff” reflected on by a student |
| Home visits | Student discusses home visits conducted on Ride-Along with community outreach partner |
| Physical exams | Student discusses physical exams performed during the rotation, practicing physical exams, conducting visual exams, or learning physical exam skills. |
| Surgery | Student discusses surgery, past or present surgery experience, or emotions regarding surgery |
| Student presentations | Student discusses presentations that were part of the rotation, either at the large municipal shelter or with a partnering middle/high school |
| Targeted consults | Student discusses a targeted consult |
| **Location** | Locations visited during the rotation |
| Large open admission municipal shelter | Student discusses the large open admission municipal shelter |
| Small open admission municipal shelter | Student discusses the small open admission municipal shelter |
| Philadelphia’s largest rescue partner and provider of low-cost basic veterinary care | Student discusses the Philadelphia’s largest rescue partner and provider of low-cost basic veterinary care |
| Community outreach organization | Student discusses the community outreach organization |
| Shelter with forensics program | Student discusses the shelter with forensics program |
| Shelter with a robust behavior program | Student discusses the shelter with a robust behavior program |
| Shelter with a low-cost clinic offering affordable care, surgery, and dentals | Student discusses the shelter with a low-cost clinic offering affordable care, surgery, and dentals |
| Shelter with a HQHV spay/neuter clinic | Student discusses the shelter with a staffed HQHV spay/neuter clinic |
| Local middle and high schools | Student discusses the local middle and high schools |
| **Shelter Medicine** | Any codes relating to the realities/core concepts of shelter medicine (not including “Syllabus”) |
| Euthanasia | Student discusses the subject of euthanasia including kill vs no kill, including phrases such as time stamped, focus on shelter |
| Limitations | Student discusses limitations of resources in the shelter setting, including comparing resources of the shelter to private practice or the University Hospital, or comparing resources between different shelters.  -Student discusses offering a spectrum of care  -Student reflects on what their recommendations would be/what they would do differently (outside of a targeted consult)  -Student discusses the limitations of law enforcement/laws/ability to convict or punish people accused of animal abuse or neglect |
| **Student perspectives** | Any codes in which students describe their perspective of a specific situation/event during the rotation |
| Beneficial experience | Student discusses an experience on the rotation was beneficial to their growth as a veterinarian, prepared them for clinical practice, the student mentions gaining confidence, or added to their skill set, or overcoming fears/anxieties |
| Changed perceptions | Student discusses a perception they had on anything, including but not exclusive to shelter medicine, that the rotation changed |
| Human-Animal Bond | Student discusses the human animal bond directly or discusses a beneficial relationship/bond a person has with an animal |
| Judgement | Student discusses judgement on low socioeconomic pet owners – including both personal judgement (students negatively judging this clientele) and mentions of others judgement (students discussing others negatively judging this cohort of clientele) |
| Takeaways from activities | Student discusses taking something new they learned from the rotation and how they plan on incorporating that into practice |
| **Syllabus** | Core topics/lessons from the course syllabus (specifically the “learning opportunities” descriptions) students discussed in their entries |
| Animal welfare/ethics | Student discusses animal welfare within or outside of rounds, well-being, a student reflects on good or poor welfare/wellbeing of an animal on rotation or outside of rotation  -Student discusses ethics, ethical dilemmas, not knowing what the right choice for an animal would be, struggling with what would be best for an animal  -Student discusses making a difficult decisions without a clear “right” answer. |
| Biosecurity | Student discusses topics related to biosecurity, public health concepts, herd health, disease outbreaks, biosecurity protocols, disease control, population control, isolation, or quarantine within or outside of rounds |
| Behavior | Student discusses behavior rounds, behavior sessions, behavior activities, or other topics related to animal behavior |
| Dental | Student discusses dental activity |
| Exotic animal | Student discusses exotics rounds, exotic animal housing, exotics in the shelter setting, exotic animal medicine |
| Community outreach | Student discusses community outreach activities, continuing education, humane education, education, outreach, any activity involving community, communication, communication skills; relaying topics for different education/knowledge/ backgrounds |
| Feral cat management (TNR/SNR) | Student discusses the topic of feral cats within or outside of animal welfare rounds, feral cat management, TNR, SNR, community cats, trapped cats |
| Humane Law | Student discusses humane law enforcement rounds, animal law enforcement, specific criminal cases, forensics, documenting an animal abuse case, holding animals for cruelty cases, animal hoarding, animal abuse, neglect, starvation, dog/cock fighting including discussion of dog/cock fighting equipment |
| Real life | Student discusses real life rounds or topics discussed during real life rounds including: compassion fatigue, burnout, managing stress, similarities between private practice and shelter medicine stressors, general stressors of the field as a whole, contract negotiation, jobs |
| Pain scoring | Student discusses pain scoring, pain scoring post-op, pain scoring development, relationship to behavior, any mention of student recognizing pain in a patient |
| Public health | Student discusses public health rounds (this is specific to these rounds) |
| Risk analysis | Student discusses risk analysis rounds, risk analysis worksheet, risk analysis chart, and webinars |
| Shelter operations | Student discusses shelter protocols, how different shelters are run/operated, how populations are managed, ASV shelter standards, or how to adapt shelter standards to facilities, what being a shelter veterinarian encompasses, facility design, kill status (“kill” vs “no kill” facilities), atmosphere of different shelters, challenges of shelters, reflecting on differences between shelters, how shelters work together |
| Spay/neuter | Student discusses spay/neuter in relation to educating and outreach |
